# A snapshot of radiation therapy techniques and technology in Queensland: An aid to mapping undergraduate curriculum

**DOI:** 10.1002/jmrs.5

**Published:** 2013-02-05

**Authors:** Pete Bridge, Mary-Ann Carmichael, Carole Brady, Allison Dry

**Affiliations:** 1 School of Clinical Sciences, Queensland University of TechnologyBrisbane, Queensland, 4001, Australia; 2 Radiation Oncology Mater CentreRaymond Terrace, South Brisbane, Queensland, 4101, Australia; 3 Cancer Care Services Royal Brisbane Women's Hospital HerstonBrisbane, Queensland, 4029, Australia

**Keywords:** Curriculum, IGRT, IMRT, radiation therapy, technology

## Abstract

**Introduction:**

Undergraduate students studying the Bachelor of Radiation Therapy at Queensland University of Technology (QUT) attend clinical placements in a number of department sites across Queensland. To ensure that the curriculum prepares students for the most common treatments and current techniques in use in these departments, a curriculum matching exercise was performed.

**Methods:**

A cross-sectional census was performed on a pre-determined “Snapshot” date in 2012. This was undertaken by the clinical education staff in each department who used a standardized proforma to count the number of patients as well as prescription, equipment, and technique data for a list of tumour site categories. This information was combined into aggregate anonymized data.

**Results:**

All 12 Queensland radiation therapy clinical sites participated in the Snapshot data collection exercise to produce a comprehensive overview of clinical practice on the chosen day. A total of 59 different tumour sites were treated on the chosen day and as expected the most common treatment sites were prostate and breast, comprising 46% of patients treated. Data analysis also indicated that intensity-modulated radiotherapy (IMRT) use is relatively high with 19.6% of patients receiving IMRT treatment on the chosen day. Both IMRT and image-guided radiotherapy (IGRT) indications matched recommendations from the evidence.

**Conclusion:**

The Snapshot method proved to be a feasible and efficient method of gathering useful data to inform curriculum matching. Frequency of IMRT use in Queensland matches or possibly exceeds that indicated in the literature. It is recommended that future repetition of the study be undertaken in order to monitor trends in referral patterns and new technology implementation.

## Introduction

Undergraduates studying the Bachelor of Radiation Therapy at Queensland University of Technology (QUT) undertake clinical placements in a variety of department sites, where they are exposed to a wide range of techniques and technologies. Although this offers numerous advantages in terms of a wide-ranging clinical experience and exposure to different equipment and procedures, there are challenges associated with ensuring undergraduates attend an appropriate mix of placements. One challenge is that placement sites differ in patient case-mix and thus in their opinion of “common” and “routine” treatments. Part of this may be due to radiation oncologist referral patterns and speciality; hence, changes in referral pattern in one centre may not necessarily be reflected in other centres. From an academic planning perspective, there is a risk that this bias in referral patterns may filter through to curriculum content if feedback is sought from individual clinical centres. A curriculum is a “dynamic process that needs to be reviewed constantly.”^1^ It is vitally important that educational curricula remain current and swiftly react to changes, particularly against the background of the rapidly evolving world of radiation therapy. While horizon-scanning reports and surveys^2^,^3^ provide useful technical alerts, more subtle changes to statewide treatment patterns and widespread use of technologies is more difficult to detect. The focus of most of these reports is on the distribution of new equipment rather than implementation and frequency of use. In order to more closely match the curriculum to statewide clinical practice requirements and better prepare the students for their supervised practice year, a data collection exercise was undertaken as a collaboration between academic staff and radiation therapy clinical educators across Queensland.

## Aims

The primary aim for the study was to collect current clinical practice information to inform curriculum mapping exercises. This was performed in order to ensure that appropriate weighting was applied to the most common treatments and most current techniques. A secondary aim was to provide a baseline of current technology usage across Queensland for future trend monitoring exercises.

## Methods

The data collection harvested quantitative patient treatment data on a single selected “Snapshot” day on 7 March 2012, across all radiation therapy clinical placement sites in Queensland. A date of convenience was chosen by the researcher without consultation from clinical departments in order to reduce selection bias. All radiotherapy providers within Queensland were chosen as these are the primary clinical placement centres for students on the QUT Course. It was important to ensure that the data reflected the range of techniques that the students would be exposed to during placements. Because of this sampling was deemed inappropriate and a census approach was utilized to collect data from all the sites. An Excel proforma was developed to enable tabulation of number of patients, prescription, and simple technique data for a range of tumour sites. The spreadsheet was populated with a draft list of 60 common tumour sites. These were determined following discussion with clinical staff from various Queensland departments. The census was performed collaboratively by the clinical educators and academic staff, and local educators were responsible for ensuring accurate data collection. Data were collected in order to provide information about the relative proportions of treatments as well as the use of more specialist technology or techniques. Data collection techniques were varied according to the systems in place locally; some centres performed manual data collation, whereas others designed database queries to collect information directly from the verification system. All data were electronically forwarded to the research lead then immediately anonymized and collated in order to generate purely accumulative quantitative information. The audit and subsequent publication was determined to be exempt from the need for University Human Research Ethics Committee review, approval, and monitoring in conformity with sections 5.1.22 and 5.1.23 of the National Statement on Ethical Conduct in Human Research. Descriptive data analysis was conducted using Excel statistical functions. After data analysis curriculum mapping was performed with the existing tumour sites, technique, planning, and technology topics being mapped from the content list of each “radiation oncology” and “radiotherapy planning” unit in the course. Since the curriculum is vertically integrated (thus understanding is developed over time), it was important to further collate elements of these topics from different parts of the course so that numbers of teaching sessions and hours dedicated to each topic could be listed. Once this process was complete, the “Snapshot” data were incorporated into the map. The aims of the mapping were to highlight gaps in the existing content material and discrepancies between the teaching hours and clinical prevalence for different oncology, technique, and planning topics.

## Results

### Demographics

All 12 Queensland radiation oncology clinical sites were able to provide data to produce a comprehensive overview of clinical practice on the chosen day. A total of 1014 patients were treated on the chosen day, including 108 patients receiving electron therapy and five undergoing kilovoltage (kV) radiotherapy.

### Most common treatment sites

A total of 59 different tumour sites were treated on the chosen day. The original list of 60 categories covered most of the different anatomical sites as well as distinguishing between radical and palliative treatments. In addition to the stated categories, an “other” category was provided. Treatment sites listed under this other category included orbit, ear, plasmacytoma, angiosarcoma, face, and squamous cell carcinomas (SCCs) of the mandible and floor of mouth. Categories that were not treated on the chosen day included liver secondary, myeloma, ocular melanoma, ovary, small bowel, spleen, testes, thymus, thyroid, trachea, and vagina. The most commonly treated sites (those with over 10 patients per day) are summarized in Table 1. As expected, breast and prostate together comprise a significant proportion (46%) of statewide workload (467 of 1014 treatments).

**Table 1:** Most common treatments

Treatment site	Number of patients
Breast tangents	187
Prostate/prostate bed	126
Prostate and seminal vesicles	83
Breast tangents and nodes	71
Lung primary	62
Skin SCC	58
Bone secondary	48
Rectum	38
Brain primary	31
Brain secondary	25
Oesophagus	21
Cervix	19
Lymphoma, non-Hodgkin	19
Larynx	17
Parotid	15
Melanoma	14
Bladder	13
Lymph nodes secondary	12
Skin BCC	12
Soft tissue sarcoma	12
Tongue	12
Tonsil	11
Uterus	11

SCC, squamous cell carcinoma; BCC, basal cell carcinoma.

### Resource use

#### Intensity-modulated radiotherapy

Data were harvested relating to use of static gantry intensity-modulated radiotherapy (IMRT), arc-based IMRT (comprising volumetric arc therapy [VMAT], RapidArc, and tomotherapy), stereotactic radiotherapy, 4DCT (four-dimensional computed tomography), and fusion technology. This information was primarily intended to provide a baseline for future comparison to show how technology use is changing across the State. Table 2 illustrates the reported use of technology on the “Snapshot” day. It can be seen that uptake of IMRT is relatively high (199 of 1014 treatments).

**Table 2:** Technology use

Technology	Number of patients, *n* (%)
4DCT planning	5 (0.5)
MR fusion	69 (6.8)
PET fusion	114 (11.2)
Static gantry IMRT	122 (12)
Arc (tomotherapy, VMAT etc)	77 (7.6)
Stereotactic radiotherapy	3 (0.3)

4DCT, four-dimensional computed tomography; MR, magnetic resonance; PET, positron emission tomography; IMRT, intensity-modulated radiotherapy; VMAT, volumetric arc therapy.

The data were further analysed to determine which categories of treatment were the prime indicators for IMRT as illustrated in Table 3. This revealed that, as expected, prostate (78) and breast (28) dominated, with paediatric, brain, and head and neck sub-categories also being typical candidates. However, when all the conventional “head and neck” sub-categories were combined into a “head and neck” category, there were 40 patients receiving IMRT, actually making this the second most common indication after prostate.

**Table 3:** IMRT and arc indications

Tumour site	Patient numbers
IMRT indications	
Prostate	50
Breast	28
Brain primary	8
Tonsil	7
Tongue	5
Larynx	4
Skin SCC	3
Oropharynx	3
Rectum	2
Cervix	2
Lymph nodes (secondary)	2
Benign tumours	2
All head and neck	20
Arc therapy indications	
Prostate	28
Tongue	5
Larynx	4
Paediatric (not CNS)	4
Brain primary	3
Tonsil	3
Skin SCC	3
Oropharynx	3
Hypopharynx	3
Cervix	2
Oesophagus	2
Whole CNS	2
All head and neck	20
Combined IMRT	
Prostate	78
Head and neck	40
Breast	28
Skin SCC	6
Paediatric	4
Cervix	4
Brain	3

IMRT, intensity-modulated radiotherapy; SCC, squamous cell carcinoma; CNS, central nervous system.

#### Imaging

Table 2 indicates that 183 (18%) patients had undergone image fusion as part of their planning process. It should be noted that these numbers relate to numbers of patients on treatment who had image fusion performed during their planning process as opposed to the number of actual fusions performed on the chosen day. Positron emission tomography (PET) fusion appears to be more widely used than magnetic resonance (MR) fusion. The indications for fusion were tabulated (Table 4) and revealed that brain and rectum were the strongest indicators for MR fusion, with lung, rectum, and oesophagus leading the PET indications.

**Table 4:** Fusion indications

Site	Patient numbers
MR fusion	
Brain primary	14
Rectum	11
Prostate	8
Brain secondary	5
Skin SCC	4
Lung primary	3
Oropharynx	3
Soft tissue sarcoma	3
Bone secondary	2
Larynx	2
Uterus	2
PET fusion	
Lung primary	31
Rectum	12
Oesophagus	12
Tonsil	7
Tongue	6
Skin SCC	5
Oropharynx	5
Larynx	4
Brain secondary	3
Hypopharynx	3
Melanoma	3
Lymphoma, NHL	3
Prostate	2
Cervix	2

MR, magnetic resonance; PET, positron emission tomography; SCC, squamous cell carcinoma; NHL, non-Hodgkin's lymphoma.

**Figure 1:**
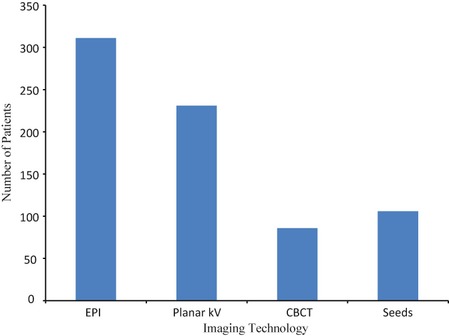
Frequency of image-guided radiotherapy technology use.

Departments were also asked to provide data concerning their use of image-guided radiotherapy technology (IGRT) including weekly and daily electronic portal images (EPI), planar kV, cone-beam computed tomography (CBCT), and fiducial markers (seeds). Figure 1 illustrates the frequency of use of these modalities. The most common imaging modalities used were clearly planar based EPI (311 patients for weekly and daily combined) and kV (231 patients) with volumetric (CBCT) being performed for 86 patient cases. Data demonstrate that there were 106 patients with implanted seeds that could be used to facilitate setup. These were almost entirely (98%) prostate patients with one secondary bone and one brain primary also having seeds.

### Curriculum mapping

Some valuable data were collected in order to perform accurate curriculum mapping and as a result of the exercise a number of key changes were made to the course curriculum as seen in Table 5.

**Table 5:** Curriculum changes

Content	Snapshot finding	Resultant change
IMRT planning	Very widespread use for prostate, breast, head and neck	Earlier introduction of IMRT technique teaching to planning units. Extended practical experience with IMRT prostate to now include breast and head and neck planning in planning units
Image fusion	Brain and rectum strong indicators for CT-MR fusion	Extension of image fusion practical experience to include CT-MR for rectum
	Lung, rectum, and oesophagus good indicators for CT-PET fusion	Extension of CT-PET fusion practical experience to include rectum and oesophagus
Arc therapy	Widespread use of volumetric arc therapy	Increased teaching related to use of arc techniques and practical planning experience
Common tumour sites	Reduced incidence of some tumour sites	Reduction in teaching hours for less common tumours and shift towards directed independent learning for these sites. Compilation of “essential” tumour list to direct student achievements

IMRT, intensity-modulated radiotherapy; CT, computed tomography; MR, magnetic resonance; PET, positron emission tomography.

## Discussion

### Impact of “Snapshot” data on curriculum

The rationale for the “Snapshot” study was primarily to inform academic curriculum planning. From an academic perspective, the data analysis has resulted in a renewed confidence in curriculum matching. The data have enabled production of an “essential tumour sites” list to focus teaching of oncology, technique, and clinical practice. In addition, this list can be used to help guide students' clinical experiences, such as choice of case studies. It is important to note that the data have been used with a significant caveat, which is that a low incidence of a technique or tumour site on the assigned day does not necessarily confirm a low overall incidence. As a result no tumour site material has been dropped from the curriculum, although the relative emphasis for different sites has been realigned. Less common sites, as identified by the “Snapshot” data, are still included in the curriculum but have a reduced teaching time commitment, relying more on self-directed learning such as online presentations and formative tests. Future iterations will be used to reinforce this newly allocated timing if low incidence of these tumour sites proves to be consistent.

One of the major outcomes from the study has been an increase of input into IMRT teaching to ensure that the range of sites used for planning experience represent clinical use of the technology. Additional IMRT planning practicals now build on existing prostate experience with new head and neck, and breast planning practicals also being incorporated. Given the relatively high incidence of arc-based IMRT techniques, student experience and teaching related to this technology has been increased. Another outcome relates to provision of PET fusion experiences to ensure that fusion of lung, rectum, and oesophagus are added to the practical experience curriculum. While it is important that students are prepared for clinical practice globally, the aim of the undergraduate course in Queensland is to better prepare students for their supervised practice year. Therefore, it is crucial that the range of experience and teaching materials available matches national and statewide practice.

Clearly, the increased teaching of these newer techniques can only take place in conjunction with a reduction in teaching elsewhere and this is always a challenge for curriculum development. “Older” techniques may still be in use clinically and remain valuable learning opportunities for students to consolidate underpinning radiotherapy principles. The challenge therefore lies with extending new content, while maintaining underpinning knowledge and understanding. This challenge is particularly prevalent in a rapidly developing profession such as radiation therapy. The approach taken with the planning topics involved changing technique teaching for some tumour sites from conventional three-dimensional (3D) conformal to IMRT. Rather than removing content, the approach concentrated on bringing the underpinning IMRT concept teaching forward and introduce IMRT techniques into some planning practicals. For the oncology teaching, no content was removed but instead the teaching approach was amended to phase out the resource intensive face-to-face approach for the less common sites in favour of directed independent learning. For all changes made to the curriculum, the distinction was made between teaching of content and learning of skills. However, content remains specific to individual tumour sites, for example, skills learnt from some planning techniques are easily transferred to others. Thus, wider implementation of IMRT planning practice did not entail reduction in teaching content but rather a change in approach to technique for some tumour sites, mirroring the change in clinical practice.

### IMRT use

The data not only provided curriculum matching information but also revealed an insight into technology implementation across the state; in particular, trends in IMRT use. IMRT is a treatment technique that can be delivered using a variety of technologies including multiple linear accelerator static fields^4^, linear accelerator based VMAT,^5^ and dedicated tomotherapy equipment.^6^

Snapshot data suggest that 20% of all Queensland radiation therapy patients receive IMRT. Of these, the greatest proportion of IMRT cases planned were prostate (39%), head and neck (20%), and breast (14%). More specifically, prostate data included all patients planned for radical radiation therapy to the prostate, prostate bed, or prostate and seminal vesicles. The “head and neck” category included primary tumours arising in the nasopharynx, hypopharynx, oropharynx, larynx, parotid, thyroid, tongue, and tonsil, while “breast” results included all patients receiving radiotherapy to breast tangential fields and breast tangents with nodal fields. The focus of this discussion is to provide comparison of the Queensland “Snapshot” results with current practice elsewhere as reported in the literature.

In a 2009 United Kingdom study,^7^ the authors reported the top three tumour types receiving IMRT: ear, nose, and throat (ENT) 36%, urological (33%), and breast (29%). Comparisons between these and “Snapshot” results are difficult and somewhat unreliable because the authors use different tumour categories and do not clarify which tumour sites are included in these categories, for example, “ENT” and “urological.” This variation in classification is a potential issue that thwarts true comparison, although it can be seen that the main indications for IMRT are broadly similar. It is interesting to note that 97% of the United Kingdom IMRT case-mix was related to these three categories, but the “Snapshot” results indicate a wider range of IMRT indications as the top three categories only comprise 73% of cases. This suggests wider uptake of the technology along with the growing clinical experience and evidence base since 2007. Also, it must be noted that much of the United Kingdom data (like the Australian Horizon-scanning reports^3^) focuses on numbers of centres utilizing IMRT as opposed to number of patients receiving it.

#### Head and neck IMRT

Snapshot data suggest that 56% of Queensland radiation therapy head and neck patients received IMRT. Contrasting this with trends in the use of IMRT in head and neck region in the United States of America, Sher et al.^8^ reported that 15% of head and neck patients were treated using IMRT in the years 2000 to 2005. However, in a broader and more generalized head and neck study, Guadagnolo et al.,^9^ using the same patient age group, data base, and year span, reported that 21.3% of patients were treated using IMRT. Guadagnolo et al. defined the cohort to include “oral cavity (including lip, tongue, floor of mouth, gum, or the other mouth), oropharynx (including tonsil), nasopharynx, hypopharynx, salivary gland, and other unspecified oral cavity and pharynx,” whereas Sher focused on non-metastatic squamous cell tumours of the head and neck including laryngeal tumours. The difference in the results of these two studies is potentially due to a difference in cohort definition. This is clearly a potential problem when attempting comparisons. This can be reduced by adhering to rigid guidelines for these definitions. Sher et al.^8^ stated that the larynx was omitted from the other study because of the perception that “patients with larynx cancer were less likely to receive IMRT.”

In relation to this study, the importance of the larynx being included in the head and neck figures is clearly demonstrated. Snapshot data suggest that radiation treatment to the larynx represents 24% of the total number of head and neck cases treated, and of these, 47% were treated with some form of IMRT. Irrelevant of difference in cohort definition, both Sher et al.^8^ and Guadagnolo et al.^9^ reported a 39–45% increase in the use of IMRT between 2000 and 2005, respectively, with both predicting that IMRT use will exceed these figures in the future. It would therefore be reasonable to suggest that, in Queensland, the use of IMRT in the head and neck region will also continue to increase in future years.

#### Prostate IMRT

The “Snapshot” results indicate that 37% of prostate tumours receiving radiotherapy underwent IMRT. It is interesting to compare this with the situation in the United States of America, where the treatment of prostate tumours using IMRT is currently the subject of much debate. Currently, there are studies^10^,^11^ and reports in the media^12^ suggesting that the rapid increase in the use of prostate IMRT is due to higher Medicare reimbursement costs as compared with other less expensive treatments, for example, conventional forward-planned radiation therapy. Although much of the support for this comes from less reliable sources, it remains clear that reimbursement has the potential to influence practice. Aside from this reason being posed as having a significant and continued effect on the uptake of prostate IMRT in the United States of America, there is considerable support for the continued use of IMRT based on evidence of reduced gastrointestinal morbidity and hip fractures and the potential for better cancer control through the delivery of higher target doses through greater field conformity.^11^,^13^,^14^ Nguyen et al.^10^ reported the rapid uptake of IMRT for men diagnosed with non-metastatic prostate cancer, with a rise from 28.7% in 2002 to 81.7% in 2005. This is substantially higher than the Queensland results and may indicate the impact of the United States of America billing system. Comparatively, Sheets et al.^13^ reported that the use of IMRT increased relative to conformal radiation therapy from 0.15% in 2000 to 95.7% in 2008. In Australia, the Royal Australian and New Zealand College of Radiologists (RANZCR) recommend that some form of IMRT is available in every radiation oncology department and is considered in the treatment of tumours involving the prostate or prostate and seminal vesicles.^3^,^5^

In conjunction with the evidence for the continued use of IMRT in the treatment of prostate tumours, the Australian Institute of Health and Welfare (AIHW) predicts that prostate cancer will remain the most common male cancer diagnosed in 2020.^15^ The AIHW also predicts that between 2011 and 2020, national prostate cancer diagnoses will continue to increase from three cases per 100,000 each year to somewhere between 164 and 200 cases per 100,000. These figures, combined with expected population changes, equate to approximately 28,000 new cases being diagnosed in 2020.^15^ Clearly, prostate radiotherapy is destined to remain one of the main uses of IMRT techniques, and it will be interesting to see how the percentage of patients with prostate cancer receiving IMRT changes in future iterations of the study.

#### Breast IMRT

In Queensland, it seems that breast IMRT is less widespread than head and neck or prostate with only 11% of this group of patients receiving treatment using this technology. IMRT is commonly used to treat patients with breast cancer in order to produce a more homogenous dose distribution, minimize rib dose, restrict lung volume and dose, and to reduce cardiac toxicity if treating the left breast.^16^ In a 2011 study focusing on trends in the use of IMRT to treat the whole breast, Smith et al.^17^ argued that more simplistic non-IMRT techniques may be sufficient, regardless of studies demonstrating that IMRT reduces acute skin reaction and improves long-term skin cosmesis.^18^ Using the same Medicare database as in the United States of America prostate studies, Smith et al.^17^ reported an increase in breast IMRT rates from 0.9% in 2001 to 11.2% in 2005. These figures cannot be used to predict local use of this technology as the United States of America reimbursement policy has arguably a strong influence on the adoption of IMRT in the treatment of breast cancer.^17^ An important distinction should perhaps be made between left and right breast IMRT use, with cardiac sparing being a strong indication for IMRT use on the left side.^19^ It would be interesting to collect data relating to IMRT for each side independently for future Snapshot analysis.

Recently, the AIHW report^15^ predicted that female breast cancer will continue to remain the most common cancer diagnosed in 2020. Additionally, breast cancer diagnoses will remain constant between 2011 and 2020 at 114 new cases per 100,000 female population. Not to be overlooked yet far less common, male breast cancer diagnoses will contribute 0.002 new cases per 100,000 of the Australian male population. Taking into account expected population growth, the number of new cases of breast cancer will continue to increase from approximately 14,000 (2011) to 17,000 (2020).^15^ When taking into consideration the known benefits of breast IMRT and national predictions in cancer incidence, it would seem reasonable to conclude that breast IMRT is most likely to be used in cases where superior dose homogeneity is critical and for left-sided disease to reduce cardiac toxicity.^19^

#### Summary of IMRT use

The purpose of this study was to evaluate local current trends in the use of available techniques and technologies so that appropriate weighting could be applied in the academic and clinical curriculum. The first iteration of the study has provided a useful baseline which forms the basis for this paper's discussion. Synthesis of the data combined with the RANCZR recommendation that some form of IMRT be available in every radiation oncology department^3^ demonstrates that IMRT is and will remain an important technology in cases where tumour size, location, adjacent organs, and dosimetry are critical for the patient to receive an optimal treatment outcome. The results of the Snapshot study would seem to indicate that IMRT use in Queensland is consistent with global data. The results also indicate that arc-based IMRT currently represent 39% of IMRT treatments; it will be interesting to repeat this in the future to identify trends in the techniques and technologies used to deliver IMRT.

### IGRT use

The data suggest that electronic portal imaging (EPI) is still the dominant technology underpinning IGRT use when compared with planar kV and CBCT. CBCT is a relatively new imaging modality and is clearly not in use at all centres. When the indications for CBCT on the studied day are examined it remains clear that most CBCT images are taken for head and neck (33%) with prostate and seminal vesicle patients (23%) and prostate only (9%) the next most common indications. It will be interesting to see how this pattern develops in future studies as CBCT is clearly growing into a more common modality.^20^ Similarly, planar kV is less well established than MV EPI. It is thus highly likely that the “Snapshot” results mirror the availability of the different modalities and it will be interesting to see how this balance changes in future iterations of the study. It would also be of value to include tomotherapy imaging as one of the categories for IGRT as this technology is increasingly becoming more widespread.

#### Head and neck IGRT

Although data from the “Snapshot” day cannot reveal long-term patterns in IGRT practice, it can provide an indication of which tumour sites are most likely to be subject to regular imaging, especially those following a daily imaging protocol. In the head and neck region, research^21^ has shown that daily imaging unsurprisingly increases accuracy and crucially allows for a reduction in planning target volume (PTV) expansion margin. “Snapshot” data suggest that clinical practice generally follows these recommendations for some sites with 100% of tongue, tonsil, hypopharynx, and nasopharynx patients being imaged on the study day. Only 8 of the 15 parotid patients and 11 of 17 larynx patients underwent imaging. This is perhaps surprising given the potential for tumour response and the normally small PTV margins for these sites but only really demonstrates that daily imaging has not been performed. This could be linked to the small number of these patients being treated with IMRT but more in-depth analysis is impossible with aggregate data. It remains clear, however, that IGRT for head and neck cancer is well established across the state.

#### Prostate IGRT

Another common indication for IGRT is prostate where decreased toxicity and potential dose escalation can be achieved,^22^ and the evidence base here is also strongly in favour of daily imaging, particularly for IMRT patients.^23^ The random positional error for these organs demands a daily IGRT protocol if small margins are used. There is evidence in particular that highlights the importance of daily imaging for seminal vesicle involved patients due to the high interfraction motion and deformation of these structures.^24^ “Snapshot” data revealed that imaging took place on the studied day for 89% of prostates and 87% of “prostate and seminal vesicle” patients. Interestingly, 19% of prostate and 15% of prostate and seminal vesicle images on the studied day were classified as weekly EPI. Again, it must be borne in mind that the data can only suggest the presence of daily imaging protocols and frequency of other protocols cannot be inferred. But certainly, it can be seen that most prostate patients are subjected to IGRT, although perhaps further study into the range of different imaging protocols is warranted.

### Snapshot methodology

A cross-sectional census without sampling was used to gain an overview of current practice. Similar methods are used for population census data collection, and it is important to acknowledge the limitations as well as the advantages of these techniques. The main advantage is the ability to gather data that are tailored to purpose. When comparing to an alternative approach such as annual reporting data, it allows for distinct and useful categories of data to be used. Attempting the same data interpretation based on International Classification of Disease (ICD) codes, for example, is fraught with difficulties relating these to radiation therapy techniques and technologies.

One concern of data collection is the impact on clinical staff and for most departments Snapshot proved to be a relatively quick and convenient method with reported times for full department data collection in the region of 4 hours. For some departments, however, this figure varied considerably according to the size of department, quality of data input, and levels of automation. Automatic data collection from verification systems and coding databases could offer more rapid outcomes in the future. However, as previously discussed, these methods may potentially limit the usefulness of the data. Another potential approach would be to gather summary data using the same categories as “Snapshot” but over an extended period such as a week, month, or calendar year. This would clearly be an extremely time consuming exercise.

The quantity of data harvested can also be a challenge. By using broad categories, the data analysis can be simplified and trends more easily identified. Even with this iteration of the tool the data analysis was a lengthy process. A balance must be struck between high enough levels of detail and ease of use. This method is ideal for relatively broad categories but the small numbers of patients falling into highly specific categories, including staging information, for example, would frustrate attempts at trend spotting. Identification of the most appropriate categories is obviously the key to a successful and appropriate census tool.

By its very nature, census data based on a single day can miss very low incidence tumour sites, and in this case, no patients were being treated for liver secondary, myeloma, ocular melanoma, ovary, small bowel, spleen, testes, thymus, thyroid, trachea, and vaginal tumours. Care must be taken to ensure that a lack of incidence on the “Snapshot” day is not taken to mean that these treatments do not occur. A potential issue could be whether the time of year affects referral rates. It is unlikely yet possible that referral rates change throughout the year and thus influence “Snapshot” data. Three approaches to this may be considered. Repeating the exercise several times throughout the year may highlight referral trends. Alternatively, maintaining the same date for future iterations will ensure that any such variations do not influence trend spotting. Finally, it may be of value to repeat the audit at two separate dates within each year. Further investigation is needed to identify if referral patterns are a significant problem.

It would be useful from an academic course planning perspective to analyse individual centre responses in order to determine how location and status of each centre affects technique and technology implementation. This would help inform student placement choices, but the decision to preserve centre anonymity means that this data cannot be analysed currently. This is a potential limitation, but it was deemed more important to gather data from all the centres than just those that did not require anonymity.

It is important to acknowledge that the curriculum planning process is best achieved via a multi-faceted approach including other methods such as multi-disciplinary consensus groups^25^ or the Delphi technique.^26^ Clearly, a cross-sectional census has proven to be a convenient method for collecting bulk data to inform clinically relevant content. Convenient data categories can be utilized to collect and analyse useful data. It is important that after recommended changes have been implemented future data collection uses identical data classifications to enable future trends in technology use to be monitored and compared.

## Conclusion

The Snapshot method proved to be a feasible and efficient method of gathering useful data to inform curriculum matching. Changes have been made to the curriculum in order to better reflect the clinical implementation of IMRT, volumetric arc therapy, and image fusion techniques. The results have also enabled improved mapping of oncology teaching emphasis to clinical practice as well as establishing a baseline with regard to treatment and technology implementation from which to monitor future trends. Further analysis of the first iteration data suggests that IMRT use in Queensland is on a par with, if not slightly ahead of, rates reported in the literature. IGRT use has also been seen to be closely aligned with recommendations from the evidence base, although this is affected by variations in technology availability. These findings from the “Snapshot” data have helped align curriculum with current clinical practice and helped academic course planners prioritise teaching resources to the most common techniques and technologies.

Future iterations of the method will distinguish between left and right breast treatments as well as sub-categorise “other” tumour sites. It will also allow tomotherapy images to be included as a separate IGRT category. Repetition of the method in the future will enable curriculum currency to be maintained as well as monitor trends in technology and technique usage. It will be particularly interesting to repeat this audit in other states and countries to provide comparative data. It is recommended that similar studies are undertaken on a regular basis to ensure that curriculum planners are provided with all the relevant information to ensure currency of content is maintained.
